# A case of pulmonary foreign body granuloma formation after transcatheter arterial chemoembolization for hepatocellular carcinoma

**DOI:** 10.1002/rcr2.863

**Published:** 2021-10-13

**Authors:** Saori Murata, Morio Nakamura, Shinji Sasada, Keisuke Kirita, Kota Ishioka, Saeko Takahashi, Yusuke Usui, Yumi Tsuchiya, Reishi Seki

**Affiliations:** ^1^ Department of Pulmonary Medicine Tokyo Saiseikai Central Hospital Tokyo Japan; ^2^ Department of Pulmonary Medicine National Hospital Organization Kanagawa Hospital Hadano Japan; ^3^ Department of Diagnostic Pathology Tokyo Saiseikai Central Hospital Tokyo Japan

**Keywords:** hepatocellular carcinoma, pulmonary foreign body granuloma, transcatheter arterial chemoembolization

## Abstract

We experienced a case of pulmonary foreign body granuloma diagnosed by bronchoscopy in a patient with multiple lung lesions after transcatheter arterial chemoembolization (TACE) for hepatocellular carcinoma. We speculate that the lesions may be caused by transarterial migration of the materials used for TACE.

## Graphical abstract

We present a case of pulmonary foreign body granuloma diagnosed by bronchoscopy in a patient with multiple lung lesions after transcatheter arterial chemoembolization for hepatocellular carcinoma.

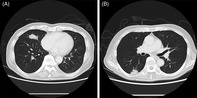

## CLINICAL IMAGE

**FIGURE 1:**
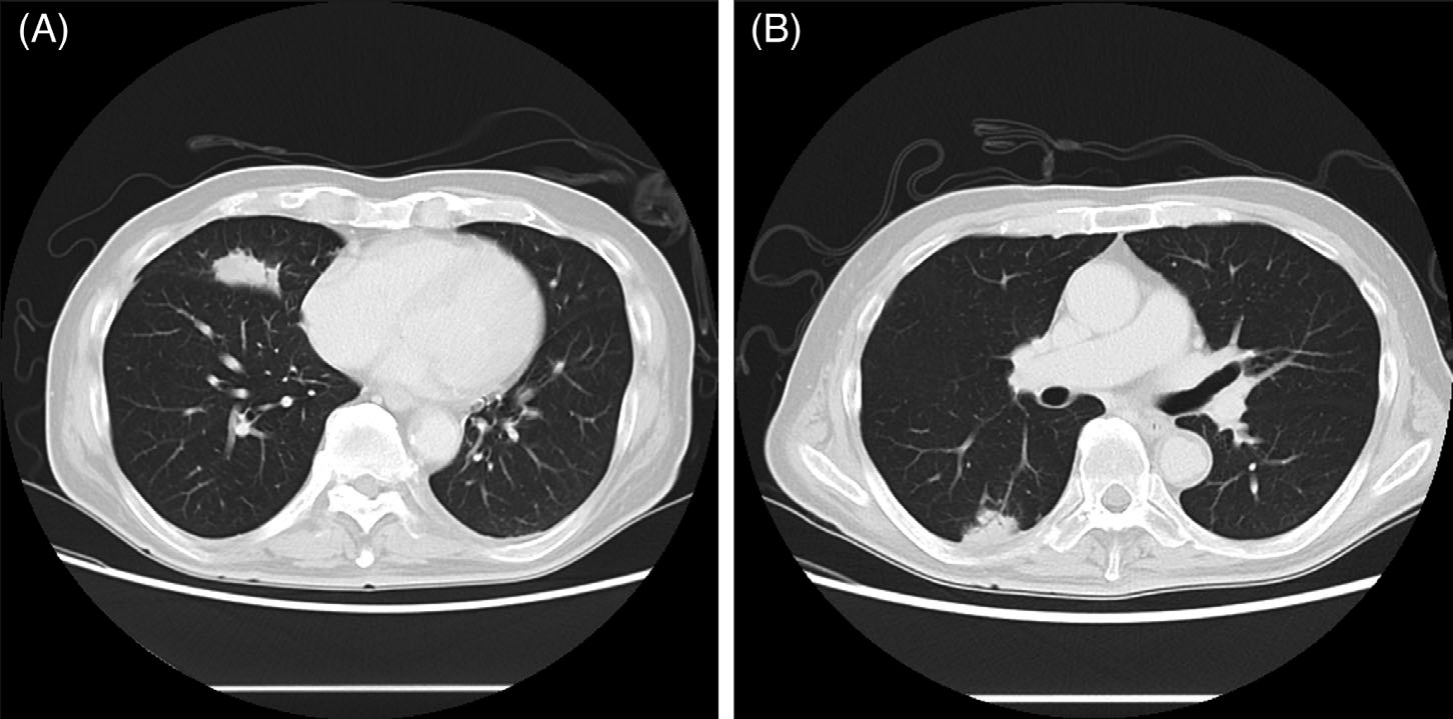
(A) Computed tomography (CT) of the chest shows an abnormal shadow in the right middle lobe 1 year after transcatheter arterial chemoembolization (TACE). (B) CT of the chest shows an abnormal shadow in the right lower lobe 3 years after TACE

**FIGURE 2:**
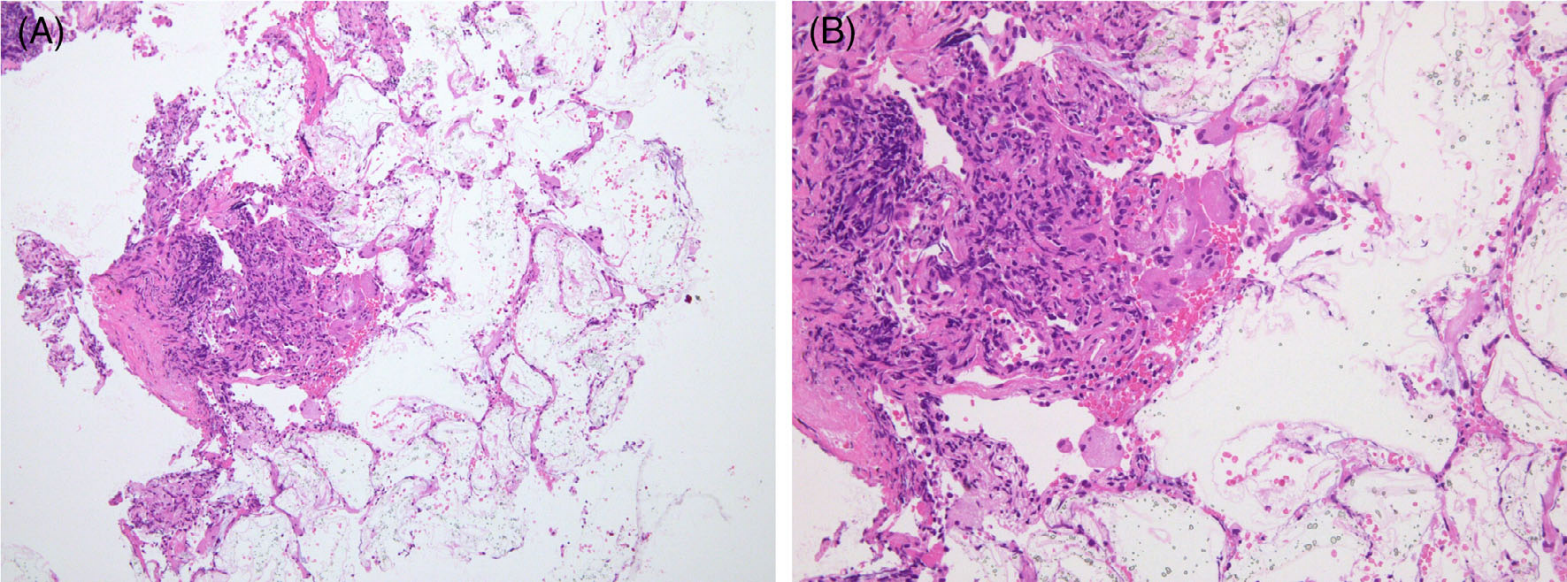
(A) At lower magnification, the alveolar structure is relatively well preserved. Pale eosinophilic unstructured material, granular material and macrophages are observed in the alveolar spaces and fibrous interstitium (haematoxylin and eosin staining, 4× magnification). (B) At higher magnification, the pale‐stained eosinophilic unstructured foreign bodies, granular lipophilic material and the surrounding foreign body‐type multinucleated giant cells are visible (haematoxylin and eosin staining, 100× magnification)

A 76‐year‐old man with no history of smoking and inhalation exposure to dust or metals underwent transcatheter arterial chemoembolization (TACE) for a recurrent hepatocellular carcinoma. In TACE, ethyl ester of iodinated poppy‐seed oil fatty acid (Lipiodol®, Guerbet Japan Co., Ltd.), gelatin sponge particles (Gelpart®, Nippon Kayaku Co., Ltd.) and epirubicin were injected into the intra‐hepatic artery. One year later, chest computed tomography (CT) revealed a thick irregular shadow in the middle lobe (Figure 1A). We performed transbronchial biopsy from the right B5a, revealing a foreign body granuloma with multinucleated giant cells (Figure 2). Three years after TACE, another lesion appeared in the right lower lobe (Figure 1B), where a second foreign body granuloma was discovered. Three months after the second biopsy, CT scan showed no obvious changes in both lesions. Pulmonary foreign body granulomatosis is a rare disorder caused by intravenous injection of crushed pharmaceutical tablets containing insoluble binding agents.^1^ In obstructed fallopian tubes, Lipiodol remains for longer than 1 year and has been reported to cause foreign body granulomas.^2^ We suggested that after TACE, these lesions can be caused by transarterial migration of chemoembolization materials from the hepatic to the pulmonary venous circulation. Long‐term follow‐up observation is however necessary for excluding pulmonary foreign body granuloma formation due to unidentified exposures in association with lesions.

## CONFLICT OF INTEREST

None declared.

## AUTHOR CONTRIBUTION

Saori Murata: conceptualization and drafting the original report. Morio Nakamura: reviewing and editing. Shinji Sasada: instructing bronchoscopy evaluation of the results, reviewing. Keisuke Kirita: instructing bronchoscopy evaluation of the results. Kota Ishioka: writing – review. Saeko Takahashi: reviewing. Yusuke Usui: supporting bronchoscopy and evaluation of the results. Yumi Tsuchiya: reviewing. Reishi Seki: pathological evaluation and diagnosis.

## ETHICS STATEMENT

The authors declare that appropriate written informed consent was obtained for the publication of this case report and accompanying images.
